# Maternal age at marriage and child nutritional status and development: evidence from Serbian Roma communities

**DOI:** 10.1017/S1368980022000544

**Published:** 2022-05

**Authors:** Jelena Čvorović

**Affiliations:** Institute of Ethnography, Serbian Academy of Sciences and Arts, Kneza Mihaila 36, Belgrade, Serbia

**Keywords:** Child marriage, Child health, Early child development, Roma

## Abstract

**Objective:**

This study aimed to assess whether maternal age at first marriage is associated with nutritional and developmental penalties in Roma children.

**Design:**

Roma nationally representative population-based study. Proxies for child nutritional outcomes included children’s individual-level height-for-age *Z* (HAZ) and weight-for-age *Z* (WAZ) scores, HAZ and WAZ scores below two standard deviations from the median of WHO’s reference population (children aged 0–59 months) and Early Child Development (ECD) (children aged 36–59 months). Multiple and logistic regressions were used to estimate the association between maternal age at marriage and the outcomes and other socio-demographic determinants as possible confounders.

**Setting:**

Aggregated data from UNICEF’s fifth and sixth Multiple Indicator Cluster Surveys for Serbian Roma settlements.

**Participants:**

Children (*n* 2652) aged 0–59 months born to ever-married women aged 15–48 years.

**Results:**

In total, 64 % of women married before age 18, 19 % of children were stunted, 9 % wasted and ECD score was low. Maternal age at first marriage was not associated with either nutritional status or early development of Roma children. Weight at birth (children aged 0–24 years) emerged as the main predictor of children’s nutritional status. Boys were more likely to be shorter, more stunted and wasted than girls. Child’s age, maternal parity and unimproved toilet facility negatively impacted nutritional status, while maternal literacy mitigated against poor nutritional and developmental outcomes.

**Conclusions:**

Roma children up to 5 years of age bear no negative consequences of maternal early marriage. The underlying determinants of children’s well-being include improved sanitation, child characteristics, maternal literacy and reproductive behavior and parental investment.

Age at marriage, being one of the most important factors in population dynamics, impacts reproductive schedule, fertility, mortality and health outcomes of both women and children and has a strong effect on a range of socio-economic, demographic and public health features^(1,2)^. Girl child marriage (first marriage before 18 years of age) is a pervasive practice not only in several former British colonies but also parts of Eastern Europe^(3–5)^. Girl child marriage and subsequent reproduction may entail health risks for both mothers and children as there is an inherent biological risk associated with young maternal age and reproductive outcomes^(6–10)^. Studies on the risks of early entry into marriage and its effects on motherhood and child health outcomes, including early development, have generated conflicting data as such risks have been shown to be mitigated by maternal, socio-economic and demographic confounding variables^(2,11–16)^. Cross-culturally the greatest risk is concentrated only in the youngest of mothers – generally those younger than 15 years, with the risk being lowest for first births^(17,18)^. Yet, despite the costs, there may also be fitness benefits associated with early reproduction, such as the advantage of longer reproductive periods and higher fertility^(19–20)^. By contrast, however, the cost of delayed onset of reproduction is associated with fewer descendants, a shorter reproductive period, longer generation times, unfavourable child outcomes, greater risk of reproductive failure and a higher cumulative mortality risk^(21–23)^.

The Roma, a diverse population of South Asian ancestry, make up the largest ethnic minority in Europe, experiencing severe social exclusion, poverty, welfare dependency and poorer health than the non-Roma^(24)^. Roma cultural traditions have encouraged division into groups based on descent alone, obligatory virginity for girls prior to their early endogamous marriages and high fertility, and thus Roma girls often enter marriage and motherhood as teenagers^(25)^. In Serbia, according to recent estimates, almost 40 % of Roma females gave birth to their first child before 18 years of age, 10 % became mothers before their 16^th^ birthday and, among women between 15 and 49 years of age, 16 % had borne a first child before age 15^(26)^. Compared with non-Roma, Roma children are often lagging in their developmental and nutritional status^(27)^. Previous research on Roma children’s health has mostly focused on social determinants of health^(28–30)^. In spite of the prevalence of child marriage and consequent early childbearing among Roma women, there is a lack of studies addressing the association between early marriage and the health outcomes of Roma children. To address this knowledge gap, the present study examined whether maternal early marriage might be associated with child nutritional and developmental penalties.

Data from the Multiple Indicator Cluster Survey rounds 5 and 6 (MICS 5 and 6) for Serbian Roma settlements were aggregated to assess the association between maternal age at marriage and child nutritional outcomes, as reflected in height-for-age *Z* (HAZ) score, stunting (HAZ –2 SD), weight-for-age *Z* (WAZ) score, wasting (WAZ –2 SD) and Early Child Development – a combined score of basic learning, socio-emotional, physical and literacy–numeracy skills.

## Method and measures

### Study sample

The present study was performed as a secondary data analysis of the MICS 5 and 6 for Serbian Roma settlements, a public use data sets, as conducted in Serbia in 2014 and 2019 and carried out by UNICEF (available at http://mics.unicef.org/surveys). The surveys provide estimates of child health indicators at the national level and separately for Roma communities. MICSs include both anthropometric and ECD data along with basic information on mothers, caregiving practices for young children and households. The surveys include questions on several domains of child development and parental engagement. Roma mothers provided information on their children’s age, gender, birth order, care and feeding practices and parental stimulating caregiving practices.

The sample included 2652 Roma children aged 0 to 59 months, born to ever-married women, aged between 15 and 48 years. Of the 2652 children, 13 % had missing data. The missing data were tested with MCAR test (Little’s Missing Completely at Random test), indicating missing at random (*χ* ^2^ (219) = 2989·98, *P* < 0·01), and estimated by separate multiple imputation regression models, including all other covariates as predictors. Statistical analyses were conducted in R (version 4.0.2, MICE version 3.11.0.).

### Measures

Maternal age at first marriage, a key exposure variable, was self-reported by Roma mothers.

### Outcome variables: child growth and development

Proxies of child nutritional outcomes included children’s individual-level HAZ and WAZ scores, and HAZ and WAZ scores below two standard deviations (–2 SD, as an indicator of stunting and wasting, respectively) from the median of WHO’s reference population. Deficits in height-for-age indicate long-term, cumulative insufficiencies of health or nutrition (stunting), while deficits in WAZ can indicate either acute or chronic inadequacies (wasting)^(27)^.

Early Child Development for children aged 36–59 months assessed the physical, learning, literacy/numeracy and socio-emotional developmental domains^(31)^. (UNICEF, 2019). A composite developmental score for Early Child Development was obtained by summation of the number of positive responses across the literacy-numeracy, social-emotional, learning and physical development domain items, with the total score ranging from 0 to 10 (UNICEF, 2015). The internal consistency of the Roma child development scale in this sample was *α* = 0·38, which was comparable with other recent studies using the same scale^(32)^.

### Covariates

To reduce the risk of confounding, additional variables accounting for maternal and child conditions were incorporated in the analyses. Maternal age at the time of the survey, maternal literacy skills and household access to improved toilet facility were used as proxies for socioeconomic status. Other maternal variables included age at first reproduction, parity, whether a mother ever had a child who later died (as a proxy for child mortality), current marriage status and parental stimulating caregiving practices (as a proxy for parental investment)^(30)^. Parental investment refers to any expenditure in terms of parental time, energy and resources used that benefit children^(33)^. Parenting investment and practices are fundamental for child development and health outcomes, such as growth and nutrition^(34,35)^.

Parental (mother and father) stimulating caregiving practices were mother-reported and refer to the types and number of activities a parent engaged in with a child within the past three days. Six activities: reading of books or looking at picture books; telling of stories to the child; counting or drawing with the child; singing songs/lullabies; taking the child outside the home into a yard or park; playing with the child are common proxies for quality parenting^(36,37)^. The total score of activities ranged from 0 to 6 points; the number of activities was categorised as low engagement (0–2 activities), moderate engagement (3–4 activities) and high engagement (5–6 activities). The internal consistency of maternal and paternal practices in this sample was *α* = 0·74 and *α* = 0·81, respectively.

Child variables included age in months and gender, to account for variation between younger and older children, and boys and girls in growth and development, child’s birth order, and weight at birth and child wantedness (whether the child was wanted at the time of conception), for children aged 0–24 months.

### Statistical analyses

Descriptive statistics were used to describe the socio-demographics of the Roma women and child anthropometric variables. Since very young age at marriage (and childbirth, i.e. <15 years) was more likely to be a risk factor for poor maternal and child outcomes^(14)^, the Roma mothers were divided into three groups based on age at first marriage: first married at 10–15, 16–17 and ≥18 years of age. These cut-off points were chosen based on the sample characteristics (i.e. the earliest age at first marriage was 10) and previous studies^(16,38)^. Chi-square, ANOVA and Tukey HSD tests and effect size were used to assess the differences in demographic and child outcomes between Roma mothers in the different marriage groups.

The sample of Roma children was divided into younger children, aged 0–35 months, and older children, aged 36–59 months. In addition, separate analyses were conducted for children aged 0–24 months, to assess the impact of birth weight and child wantedness on the outcomes. There were thus 1033 children aged 0–24 months, 1577 children aged 0–35 and 1075 children aged 36–59 months.

To assess the relationship between maternal age at first marriage and Roma children’s individual-level HAZ and WAZ scores (for children aged 0–24, 0–35 and 36–59 months), and between age at first marriage and children’s early development (for children aged 36–59 months), several multiple linear regressions were conducted. In addition, to assess the association between age at first marriage and children’s stunting and wasting, separate logistic regressions were performed for children in the three age groups.

All regressions included a series of models: the first, unadjusted model explored the association of the exposure (age at first marriage) with each outcome. The second model controlled for biological and reproductive variables: maternal age, age at first reproduction, parity, child mortality, child’s age, gender, birth order and weight at birth and child wantedness, where available. The third model incorporated proxies for socio-economic status: maternal literacy and access to improved toilet facility. The fourth, a fully adjusted model, included home environment, such as maternal marriage status, and parental investment. Only full models are presented.

In the multiple regressions, the dependent variables, i.e. individual-level HAZ and WAZ and ECD scores were continuous, while in the logistic regressions HAZ and WAZ scores were dichotomous and coded as 0- <-2 SD and 1- >-2 SD. Maternal age, age at first marriage and first reproduction, child’s age, weight at birth and birth order were continuous variables, while marriage status (0-not in a union, 1-in a union), child wantedness (0-unwanted, 1-wanted), child gender (1-boys, 0-girls), child mortality (1-yes, 0- no), maternal literacy (0-illiterate, 1-literate), type of toilet facility (0-unimproved, 1-improved) were dichotomous. Parental stimulation was continuous in multiple and categorical (low, moderate and high engagement) in logistic regressions. All predictor variables were standardised prior to regressions, except categorical parental stimulation.

## Results

The average age at first marriage for Roma mothers was 17 (range 10–37, M_d_ = 17, IQR in between 15 and 19 years of age, Q1 = 15 and Q3 = 19, not shown), while age at first reproduction was 18 (range 12–38). About 35 % of mothers married between the ages of 10–15, 29 % married between 16–17 years of age, while 36 % percent married at ≥18. Thus, 64 % of Roma women married before age 18.

Table 1 summarises the demographics, children’s nutritional status and development and differences by mother’s age at first marriage (married at: 10–15, 16–17 and ≥18 years of age) for the Roma mothers and children.

**Table 1:** Demographics, children’s nutritional status and development and differences by mother’s age at first marriage (married at: 10–15, 16–17 and ≥18 years of age) for Roma mothers and children

Marriage groups	Pooled data						*P* *	Whole sample		
	10–15 *n* 915·05		16–17 *n* 770·55		≥18 *n* 966·4			original data		*n*
	*n*	%	*n*	%	*n*	%		*n*	%	
Age at marriage								17·09	3·34	2482
Biological and reproductive variables										
	Mean	sd	Mean	sd	Mean	sd		Mean	sd	
Mother’s age	23·51	5·49	24·83	5·17	28·22	5·53	<0·001‡	25·59	5·79	2505
Parity	3·33	1·77	3·01	1·59	2·83	1·59	<0·001‡	3·04	1·67	2505
Child mortality										
No	872·45	95·34	746·35	96·86	933·80	96·63	0·077†	96·2		2411
Yes	42·60	4·66	24·20	3·14	32·60	3·37		3·8		94
	Mean	sd	Mean	sd	Mean	sd		Mean	sd	
Age at first reproduction	15·82	1·82	17·87	2·04	21·27	3·03	<0·001‡	18·43	3·34	2497
Child characteristics										
Child’s age	29·98	16·91	29·77	16·76	30·60	17·36	0·438‡	30·23	17·28	2564
Gender										
Male	472·30	51·61	397·90	51·64	502·80	52·03	0·725†	51·8		1373
Female	442·75	48·39	372·65	48·36	463·60	47·97		48·2		1279
	Mean	sd	Mean	sd	Mean	sd		Mean	sd	
Birth order	2·91	1·78	2·58	1·58	2·49	1·56	<0·001‡	2·66	1·65	2504
Weight at birth	3·06	0·50	3·02	0·55	3·02	0·56	0·302‡	3·04	0·54	984
Child wantedness										
No	345·35	95·82	297·75	98·01	356·65	96·71	0·112†	15·9		159
Yes	15·05	4·18	6·05	1·99	12·15	3·29		84·1		842
	Mean	sd	Mean	sd	Mean	sd		Mean	sd	
Height for age *Z*-score	−1·06	1·30	−1·02	1·34	−0·82	1·38	<0·001‡	−0·95	1·34	2301
Height for age *Z*-score WHO										
<-2 SD	193·45	21·14	151·70	19·69	178·10	18·43	0·146†	19·4		446
>-2 SD	721·60	78·86	618·85	80·31	788·30	81·57		80·6		1855
	Mean	sd	Mean	sd	Mean	sd		Mean	sd	
Weight for age *Z*-score WHO	−0·59	1·16	−0·62	1·11	−0·41	1·20	<0·001‡	−0·52	1·16	2367
Weight for age *Z*-score WHO										
<-2 SD	96·95	10·60	69·75	9·05	78·30	8·10	0·068†	9·1		216
>-2 SD	818·10	89·40	700·80	90·95	888·10	91·90		90·9		2151
	Mean	sd	Mean	sd	Mean	sd		Mean	sd	
Early child development score	1·57	1·39	1·55	1·37	1·65	1·30	0·452‡	1·59	1·35	1028
Socio-economic status										
Basic literacy										
Illiterate	339·05	37·05	227·05	29·47	319·55	33·07	0·001†	33·7		749
Literate	576·0	62·95	543·5	70·53	646·85	66·93		66·3		1472
Type of toilet facility										
Unimproved and open	195·85	21·40	113·15	14·68	132·90	13·75	<0·001†	16·7		441
Improved	719·20	78·60	657·40	85·32	833·50	86·25		83·3		2200
Home environment										
Marital status										
Not in union	71·05	7·76	81·05	10·52	100·65	10·41	0·027†	9·6		241
Currently married or living with man	844	92·24	689·50	89·48	865·75	89·59		90·4		2264
	Mean	sd	Mean	sd	Mean	sd		Mean	sd	
Maternal investment	2·48	1·86	2·63	1·95	2·81	2·00	0·001‡	2·54	1·94	
Maternal investment										
Low	248·60	27·17	202·50	26·28	231·90	23·99	<0·001†	25·8		683
Moderate	532·15	58·16	426·35	55·33	519·50	53·76		55·7		1478
High	134·30	14·68	141·70	18·39	215·00	22·25		18·5		491
	Mean	sd	Mean	sd	Mean	sd		Mean	sd	
Paternal investment	1·26	1·43	1·31	1·47	1·45	1·61	0·006‡	1·34	1·51	
Paternal investment										
Low	426·8	46·64	344·45	44·70	410·75	42·50	0·002†	44·6		1182
Moderate	458·8	50·14	395·9	51·38	498·3	51·56		51·0		1353
High	29·45	3·22	30·2	3·92	57·35	5·94		4·4		117

* *P* = ≤ 0·05.

† *χ* ^2^ test performed.

‡ ANOVA performed.

Roma mothers were relatively young, with an average age of 25 (M_d_ = 24, IQR in between 21 and 29 years of age, Q1 = 21 and Q3 = 29, not shown), with around 34 % being illiterate. At the time of the surveys, most women were living in a union. Around 17 % lived in household without access to improved sanitation (toilet facility). Parental investment was low, but higher for mothers. The average number of children was 3 (range 1–11), with an excess of males. The average age of the children was 30 months. For the children weighed at birth (0–24 months old), the average weight was 3 kg, with over 15 % being born with low birth weight (≤2·5 kg). About 19 % of children were stunted, 9 % wasted, and for children aged 36–59 months, the Early Child Development score was low (2 out of 7).

Roma mothers who married at ages 10–15 were the youngest (F_1,1934·78_ = 320·89, *P* < 0·01, moderate size effect, *η* ^2^ = 0·12; married at 10–15 (M = 23·51, sd = 5·49) *v*. ≥18 (M = 28·22, sd = 5. 53), *P* < 0·01; married at 10–15 (M = 23·51, sd = 5·49) *v*. 16–17 (M = 24·83, sd = 5·17), *P* < 0·01), while those married at ≥18 were the oldest in the sample (married at ≥18 (M = 28·22, sd = 5·53) *v*. 16–17 (M = 24·83, sd = 5·17), *P* < 0·01). Women who married at 10–15 had higher parity than women in the two other groups (F_1,2963·07_ = 39·49, *P* < 0·01, small size effect, *η* ^2^ = 0·02; married at 10–15 (M = 3·33, sd = 1·77) *v*. ≥18 (M = 2·83, sd = 1·59), *P* < 0·01, 10–15 (M = 3·33, sd = 1·77) *v*. 16–17 (M = 3·01, sd = 1·59), *P* < 0·01), while those married at ≥18 had the lowest parity (≥18 (M = 2·83, sd = 1·59) *v*. 16–17 (M = 3 01, sd = 1·59), *P* = 0·04). The 10–15 group of women had an earlier age at first reproduction when compared with the others (F_1,1114·7_ =2105·90, *P* < 0·01, large size effect, *η* ^2^ = 0·48; married at 10–15 (M = 15·82, sd = 1·82)) *v*. ≥18 (M = 21·27, sd = 3·03), *P* < 0·01, 10–15 (M = -15·82, sd = 1·82)) *v*. 16–17 (M = 17·87, sd = 2·04), *P* < 0·01), while those married at ≥18 reproduced at the latest age (≥18 (M = 21·27, sd = 3·03) *v*. 16–17 (M = 17·87, sd = 2·04), *P* = 0·04). Illiteracy was the highest among women married at 10–15 years of age (*χ* ^2^ (1, 2624·34) = 10·17, *P* < 0·01), they had less access to improved toilet facility (*χ* ^2^ (1, 7900·19) = 21·81, *P* < 0·01) and were least likely to be single (*χ* ^2^ (1, 7457·51) = 4·92, *P* = 0·03) compared with the others. Also, women who married at 10–15 and their partners provided less parental investment compared with women who married at ≥18 and their partners (F_1,467·90_ = 10·94, *P* < 0·01, small size effect, *η* ^2^ = 0·01, *t* (480·79) = 3·32, *P* < 0·01) and *χ* ^2^ (1, 4370·05) = 17·35, *P* < 0·01); (F_1,4726·60_ = 7·51, *P* = 0·01, small size effect, *η* ^2^ = 0·00, *t* (4657·55)= 7·73, *P* = 0·01 and *χ* ^2^ (1, 6001·16) = 10·06, *P* < 0·01, respectively).

Children born to mothers who married at ages 10–15 had higher birth order than children born to mothers who married at ≥18 and 16–17 years of age (F_1,3689·74_ = 27·62, *P* < 0·01, small size effect, *η* ^2^ = 0·01, 10–15 *v*. ≥18, *P* < 0·01, and 10–15 *v*. 16–17, *P* < 0·01), were shorter than children born to mothers who married at ≥18 (F_1,1560·41_ = 13·56, *P* < 0·01, small size effect, *η* ^2^ = 0·01, 10–15 *v*. ≥18, *P* < 0·01), who, in turn, had taller children than mothers married at 16–17 years of age (*P* < 0·01). Mothers who married at ages 10–15 had lighter children than mothers who married at ≥18, whose children were heavier than those born to mothers who married at 16–17 (F_1,2213·56_ = 11·28, *P* < 0·01, small size effect, *η* ^2^ = 0·01, *P* < 0·01 and *P* < 0·01, respectively).

Weight at birth, stunting, wasting and early development did not differ between the Roma children (*P* > 0·05).

Table 2 summarises the results of the multiple linear regressions to assess the relationship between maternal age at first marriage and Roma children’s individual-level HAZ scores, and logistic regressions to assess the relationship between maternal age at first marriage and Roma children’s stunting (−2 SD HAZ), per each age group (children aged 0–24, 0–35 and 36–59 months).

**Table 2:** Multiple linear regression analyses: associations of maternal age at first marriage and Roma children individual-level height-for-age *Z* (HAZ) scores and logistic regression analyses: associations of maternal age at first marriage and Roma children stunting (−2 SD HAZ)

	HAZ scores						Stunting					
	0–24		0–35		36–59		0–24		0–35		36–59	
	*β*	95 % CI	*β*	95 % CI	*β*	95 % CI	OR	95 % CI	OR	95 % CI	OR	95 % CI
Age at marriage	0·08	−0·08, 0·23	0·10	−0·02, 0·22	0·01	−0·11, 0·13	1·08	0·82, 1·42	1·20	0·95, 1·51	1·17	0·90, 1·54
Biological and reproductive variables												
Mother’s age	0·14	−0·07, 0·36	0·10	−0·06, 0·26	0·11	−0·06, 0·27	1·25	0·80, 1·95	1·39	0·99, 1·95	1·18	0·82, 1·71
Parity	0·26	−0·34, 0·87	−0·24	−0·56, 0·09	−0·20*	−0·39, −0·002	0·87	0·32, 2·36	0·60	0·34, 1·04	0·92	0·59, 1·43
Child mortality												
No							Reference		Reference		Reference	
Yes	0·37	−0·17, 0·92	0·35	−0·08, 0·79	−0·14	−0·51, 0·22	1·05	0·87, 1·25	1·08	0·92, 1·26	1·02	0·89, 1·20
Age at first reproduction	−0·10	−0·28, 0·08	−0·11	−0·25, 0·04	−0·01	−0·14, 0·13	0·75	0·53, 1·07	0·67*	0·50, 0·89	0·78	0·58, 1·05
Child’s age	−0·60*	−0·97, −0·24	−0·42*	−0·61, −0·23	−0·31*	−0·50, −0·12	0·50	0·23, 1·10	0·69*	0·48, 0·98	1·80*	1·18, 2·74
Gender												
Female							Reference		Reference		Reference	
Male	−0·17*	−0·26, −0·08	−0·10*	−0·17, −0·03	0·02	−0·06, 0·09	0·70*	0·59, 0·84	0·79*	0·69, 0·91	1·01	0·85, 1·19
Weight	0·38*	0·28, 0·47					1·64*	1·38, 1·94				
Child wantedness												
No							Reference					
Yes	0·002	−0·26, 0·26					1·02	0·85, 1·21				
Birth order	−0·52	−1·15, 0·12	0·03	−0·32, 0·38	0·01	−0·23, 0·25	0·78	0·27, 2·26	1·09	0·58, 2·03	0·82	0·48, 1·41
Socio-economic status												
Basic literacy												
Illiterate							Reference		Reference		Reference	
Literate	0·08	−0·02, 0·17	0·09*	0·01, 0·17	0·14*	0·07, 0·23	1·04	0·87, 1·25	1·08	0·94, 1·25	1·22*	1·03, 1·46
Type of toilet facility												
Unimproved and open							Reference		Reference		Reference	
Improved	−0·01	−0·10, 0·08	−0·003	−0·08, 0·07	0·04	−0·04, 0·11	1·01	0·85, 1·20	0·97	0·84, 1·12	1·05	0·90, 1·23
Marital status												
Not in union,							Reference		Reference		Reference	
Currently married or living with man	0·07	−0·25, 0·40	0·17	−0·10, 0·44	0·17	−0·12, 0·45	0·99	0·83, 1·18	1·08	0·94, 1·23	1·04	0·87, 1·26
Maternal investment	0·03	−0·05, 0·11	0·01	−0·04, 0·06	0·02	−0·03, 0·07						
Paternal investment	−0·03	−0·13, 0·06	−0·002	−0·07, 0·06	0·04	−0·02, 0·09						
Maternal investment low							Reference		Reference		Reference	
Maternal investment moderate							1·04	0·50, 2·15	0·95	0·61, 1·47	1·03	0·54, 1·95
Maternal investment high							1·06	0·45, 2·51	1·19	0·68, 2·07	1·29	0·61, 2·75
Paternal investment low							Reference		Reference		Reference	
Paternal investment moderate							1·11	0·65, 1·88	0·99	0·67, 1·47	1·20	0·79, 1·83
Paternal investment high							0·67	0·18, 2·54	1·44	0·61, 3·40	1·73	0·68, 4·37

* *P* = ≤ 0·05.

### Individual-level height-for-age *Z* scores

For children aged 0–24 months, age and gender (male) were both negatively associated with height (*β* = -0·60; 95 % CI (−0·97, −0·24); *P* < 0·01; and *β* = -0·17; 95 % CI (−0·26, −0·08); *P* < 0·01, respectively), while there was a positive association with weight at birth (*β* = 0·38; 95 % CI (0·28, 0·47); *P* < 0·01). Thus, older Roma children lagged behind for 0·60 sd in regard to their age-reference group compared with younger children, Roma boys were on average shorter for 0·17 sd than Roma girls, while increase in weight at birth was accompanied by an increase of 0·38 sd in height.

For children aged 0–35 months, maternal age at marriage was significant and negatively associated with height in the first, unadjusted, model (*β* = −0·03; 95 % CI (−0·07, −0·001); *P* = 0·04): an increase in maternal age at first marriage for 1 sd was associated with a decrease in children’s height for 0·03 sd (not shown). After controlling for other maternal and child characteristics in the full model, this significance was lost. Instead, child’s age and gender were negatively associated with height, while maternal literacy was positively associated with height. Thus, an increase in child age was associated with a decrease in height for 0·42 sds (*β* = −0·42; 95 % CI (−0·61, −0·23); *P* < 0·01)), boys were on average shorter than girls for 0·10 sds (*β* = −0·10; 95 % CI (−0·17, −0·03); *P* = 0·01), while children born to literate mothers were on average taller for 0·09 sds than children of illiterate mothers (*β* = 0·09; 95 % CI (0·01, 0·17); *P* = 0·02).

For children aged 36–59 months, maternal age at marriage was statistically significant and negatively associated with children’s height only in the first model: increased maternal age at first marriage for 1 sd was associated with a decrease of children’s height for 0·02 sds (*β* = −0·02; 95 % CI (−0·05, −0·002); *P* = 0·03). Once other child and maternal variables were introduced, age at marriage became statistically insignificant. Instead, there was a negative association of child’s age and maternal parity and a positive association of maternal literacy and children’s height. Thus, an increase in child’s age was associated with a decrease in height for 0·31 sds (*β* = −0·31; 95 % CI (−0·50, −0·12); *P* < 0·01), an increase in maternal parity was associated with a decrease in height for 0·02 SDs (*β* = −0·20; 95 % CI (−0·39, −0·002); *P* = 0·04), while children born to literate mothers were, on average, taller for 0·14 sds compared with children born to illiterate mothers (*β* = 0·14; 95 % CI (0·07, 0·23); *P* < 0·01).

### Stunting (−2 sd height-for-age *Z*)

For children aged 0–24 months, boys were more likely to be stunted than girls (OR = 0·70; 95 % CI (0·59, 0·84); *P* < 0·01), while children born heavier at birth were less likely to be stunted (OR = 1·64; 95 % CI (1·38, 1. 94); *P* < 0·01).

For children aged 0–35 months, there was a negative association of maternal age at first reproduction (OR = 0·67; 95 % CI (0·50, 0·89); *P* = 0·01), child age (OR = 0·69; 95 % CI (0·48, 0·98); *P* = 0·04) and gender (OR = 0·79; 95 % CI (0·69, 0·91); *P* < 0·01) with children’s stunting. Thus, for children born to mothers with later age at reproduction, boys and older children were more likely to be stunted than their counterparts.

For children aged 36–59 months, older children with literate mothers were less likely to be stunted (OR = 1·80; 95 % CI (1·18, 2·74); *P* = 0·01, and OR = 1·22; 95 % CI (1·03, 1·46); *P* = 0·02, respectively) than their counterparts.

Table 3 presents a summary of the multiple linear regressions assessing the relationship between maternal age at first marriage and Roma children’s individual-level-weight-for age *Z* (WAZ) (per each age group), and Early Child Developmental scores (for children aged 36–59 months), and the logistic regressions accounting for the relationship between maternal age at first marriage and Roma children’s wasting (per each age group).

**Table 3:** Multiple linear regression analyses: associations of maternal age at first marriage and Roma children individual individual-level-weight-for age *Z* (WAZ) and early child developmental scores, and logistic regression analyses: associations of maternal age at first marriage and Roma children wasting (−2 SD WAZ)

	WAZ scores						ECD score		Wasting					
	0–24		0–35		36–59		36–59		0–24		0–35		36–59	
	*β*	95 % CI	*β*	95 % CI	*β*	95 % CI	*β*	95 % CI	OR	95 % CI	OR	95 % CI	OR	95 % CI
Age at marriage	0·01	−0·10, 0·13	0·04	−0·06, 0·14	0·05	−0·07, 0·16	−0·04	−0·18, 0·09	0·95	0·64, 1·41	1·00	0·74, 1·34	1·22	0·84, 1·76
Biological and reproductive variables														
Mother’s age	0·09	−0·08, 0·26	0·07	−0·06, 0·20	0·13	−0·02, 0·29	−0·04	−0·21, 0·13	1·46	0·81, 2·62	1·52	0·98, 2·35	1·32	0·75, 2·33
Parity	0·04	−0·46, 0·54	−0·13	−0·41, 0·15	−0·04	−0·23, 0·15	−0·14	−0·35, 0·08	1·23	0·23, 6·64	0·90	0·39, 2·07	1·21	0·64, 2·30
Child mortality														
No									Reference		Reference		Reference	
Yes	0·24	−0·18, 0·66	0·31	−0·04, 0·66	0·12	−0·23, 0·48	0·24	−0·18, 0·65	0·98	0·79, 1·22	1·03	0·86, 1·24	0·96	0·78, 1·18
Age at first reproduction	−0·02	−0·17, 0·12	−0·04	−0·17, 0·08	−0·02	−0·15, 0·10	0·04	−0·11, 0·19	0·91	0·55, 1·49	0·85	0·58, 1·24	0·71	0·47, 1·07
Child’s age	0·28	−0·02, 0·58	−0·04	−0·20, 0·12	0·13	−0·05, 0·31	0·37*	0·16, 0·58	1·41	0·48, 4·11	0·75	0·47, 1·20	1·41	0·76, 2·62
Gender														
Female									Reference		Reference		Reference	
Male	−0·09*	−0·16, −0·01	−0·04	−0·10, 0·02	0·06	−0·01, 0·13	0·02	−0·06, 0·11	0·78*	0·63, 0·98	0·87	0·73, 1·03	1·10	0·86, 1·41
Weight	0·34*	0·26, 0·41							2·01*	1·61, 2·51				
Child wantedness														
No									Reference					
Yes	0·08	−0·12, 0·28							1·11	0·89, 1·38				
Birth order	−0·21	−0·73, 0·31	−0·02	−0·33, 0·28	−0·10	−0·33, 0·13	0·04	−0·22, 0·31	0·58	0·10, 3·30	0·77	0·31, 1·89	0·55	0·25, 1·23
Socio-economic status														
Basic literacy														
Illiterate									Reference		Reference		Reference	
Literate	0·62	−0·02, 0·14	0·06	0·00, 0·13	0·10*	0·02, 0·18	0·05	−0·04, 0·14	1·11	0·87, 1·41	1·10	0·92, 1·31	1·00	0·77, 1·29
Type of toilet facility														
Unimproved and open									Reference		Reference		Reference	
Improved	0·05	−0·03, 0·12	0·04	−0·02, 0·10	0·12*	0·05, 0·19	−0·06	−0·14, 0·03	1·10	0·89, 1·35	1·05	0·87, 1·24	1·13	0·91, 1·41
Home environment														
Marital status														
Not in union,									Reference		Reference		Reference	
Currently married or living with man	−0·01	−0·26, 0·24	0·06	−0·16, 0·27	−0·001	−0·26, 0·26	0·10	−0·21, 0·40	1·00	0·81, 1·23	1·04	0·87, 1·24	0·99	0·75, 1·30
Maternal stimulation	−0·003	−0·07, 0·06	0·04	0·00, 0·09	0·01	−0·03, 0·06	0·06*	0·01, 0·12						
Paternal stimulation	−0·02	−0·09, 0·07	0·001	−0·05, 0·05	0·04	−0·01, 0·09	−0·02	−0·08, 0·04						
Maternal stimulation low									Reference		Reference		Reference	
Maternal stimulation moderate									0·96	0·36, 2·62	1·51	0·82, 2·76	1·53	0·66, 3·54
Maternal stimulation high									0·66	0·20, 2·16	1·91	0·89, 4·08	2·33	0·83, 6·54
Paternal stimulation low									Reference		Reference		Reference	
Paternal stimulation moderate									2·13	0·97, 4·64	1·21	0·71, 2·06	1·27	0·70, 2·29
Paternal stimulation high									0·90	0·16, 5·18	0·75	0·28, 2·03	1·33	0·35, 5·04

* *P* = ≤ 0·05.

### Individual-level-weight-for age *Z* scores

For the youngest children (aged 0–24 months), gender (*β* = −0·09; 95 % CI (−0·16, −0·01); *P* = 0·02) and weight at birth (*β* = 0·33; 95 % CI (0·26, 0·41); *P* < 0·01) were associated with weight: thus boys were on average lighter for 0·09 sd than girls, while an increase in weight at birth was associated with an increase in weight of 0·33 sd.

For children aged 0–35 months, no variable influenced children WAZ (*P* > 0·05).

For children aged 36–59 months in the adjusted, full model, there was a positive association of maternal literacy (*β* = 0·10; 95 % CI (0·02, 0·18); *P* = 0·01) and the type of toilet facility (*β* = 0·12; 95 % CI (0·05, 0·19); *P* < 0·01) with weight: children with literate mothers were heavier for 0·10 sd than their counterparts, while children living in households with access to improved toilet facility were heavier for 0·12 sd than children using unimproved toilet.

### Early child development for children aged 36–59 months

In the adjusted, full model, child’s age (*β* = 0·37; 95 % CI (0·16, 0·58); *P* < 0·01) and maternal investment (*β* = 0·06; 95 % CI (0·01, 0·12); *P* = 0·03) were positively associated with early development: increase in child age for 1 sd was associated with increase in developmental score of 0·37 sds, while increase in maternal investment for 1 sd was associated with increase in developmental score of 0·06 sds.

### Wasting (−2 SD WAZ)

For children aged 0–24 months, boys were more likely to be wasted than girls (OR = 0·79; 95 % CI (0·63, 0·98); *P* = 0·04), while children with greater weight at birth were less likely to be wasted (OR = 2·01; 95 % CI (1·61, 2·51); *P* < 0·01).

For children aged 0–35 and 36–59 months, no variable influenced children wasting (*P* > 0·05).

## Discussion

This study tested for possible associations between maternal age at first marriage, child nutritional status and early development among the Serbian Roma. The majority of Roma women were ‘child brides’, i.e. they were married before age 18. In spite of the maternal early age at marriage, Roma children bore no negative consequences in terms of worse nutritional outcomes or compromised development. After controlling for maternal and child factors, maternal age at first marriage was not associated with either the nutritional status or early development of Roma children. Nor was there such association present after comparing children among women from the different marriage groups as there were no differences in children’s weight at birth, stunting, wasting or development. These findings are in line with other studies where different maternal characteristics were found to account for children’s poor nutrition and development^(10,12–14,39)^. Maternal age at marriage was statistically significant only in the unadjusted models for the HAZ scores for children aged 0–35 and 36–59 months. Unlike in other studies, these associations were negative, i.e. an increase in maternal age at first marriage was associated with a decrease in children’s height^(16,40–41,6)^.

Among the Roma, as in many traditional societies, childbirth usually follows soon after marriage, with the estimated optimal maternal age for first reproduction at 18 years^(8)^. In this sample, the observed mean age at first marriage was 17, while the mean age at first reproduction was at an optimum of 18 years. Previous studies on the Roma reproductive pattern found that Roma women start reproducing at the optimum age, continue having children in their most fertile years and cease reproduction relatively early, with later age at first reproduction being significantly associated with infant low birth weight^(20,30)^. The risks associated with later maternal age at first reproduction include changes in children’s quantity but also quality, suggesting a significant disadvantage of delayed age at first reproduction^(21,42–44)^. Thus, the association of maternal age at marriage and children’s height most likely reflected the relationship between advanced maternal age and child outcomes^(45)^, in agreement with the finding in the present sample of a negative relationship between maternal age at first reproduction and stunting in children aged 0–35 months.

Birth weight has been found in many studies to be the most important predictor of stunting among children^(46–47)^. MICSs did not collect birth weight data for all children, but where available (for children aged 0–24 months) weight at birth emerged as the main predictor of children’s nutritional status, as it could explain the HAZ and WAZ scores, stunting and wasting. Weight at birth, therefore, has emerged as one of the key specific correlates of child growth in respect of both height and weight^(46,48)^. Birth weight thus reflects maternal size, reproductive strategy and environmental limitations and may be the most important determinant of a child’s growth in early life^(49–51)^.

Child age is a further factor that was found to influence the children’s health: across all ages, older children were shorter, while for the younger group, more likely to be stunted than their counterparts. Many risk factors have been identified for growth faltering, such as maternal nutrition deficiency and infections, as well as biological factors as maternal height and fetal growth restrictions^(52–53)^. For Roma children, as for many other children living in low-income and middle-income countries, a decrease in mean HAZ with age may be due to a plunging shift of the entire HAZ distribution, suggesting that children across the HAZ range may experience slower growth compared with international norms^(54)^. Roma children have a mean HAZ of less than 0 at birth (−0·95), and the results suggest a decrease in mean HAZ in the early stages and continuing throughout the early years of life. However, the opposite trend was demonstrated in children aged 36–59 months, where older children had lower odds of being stunted. In poor populations, later-born children are often disadvantaged relative to earlier-born in nutritional status, having higher morbidity and mortality^(55)^. Maternal depletion and childbearing patterns such as closed birth spacing and inadequate diet may cause poor maternal health and poor child outcomes, especially for the youngest children^(56–57)^. However, in poor environmental and high fertility settings such as for the Roma, all children may be at risk for growth owing to a discrepancy between family size and available resources^(58)^. Parity reflects a fundamental trade-off between number and size of offspring^(59,60)^, as shown in the present study by the negative association between maternal parity and children’s HAZ scores for the oldest group of Roma children, and in the lower HAZ and WAZ scores for children whose mothers married at ages 10–15, as they had greater parity compared with the others^(61)^.

In some settings, undernutrition in children under 5 is more likely to affect boys than girls, as a combination of both biological and social mechanisms responsible for the differences^(62–64)^. Gender differences in nutritional status among the Roma children were noticeable with boys being shorter, likely more stunted, on average lighter than the girls and also more wasted (in children 0–24 and 0–35 months). These results suggest that Roma boys were more susceptible to nutritional inequalities than girls of the same age, likely implying a gender preference in favour of the girls^(65)^.

Numerous studies have shown that various indicators of socio-economic status are associated with a child’s nutritional status, e.g. maternal education and household wealth^(28,66,67)^. Roma children born to literate mothers and living in households with access to improved toilet facility were better nourished than their counterparts. Generally, Roma women spend very little time in formal schooling; in the present sample, over 30 % could neither read nor write. The costs of illiteracy and low education can be high, especially for females, as even a small increase in maternal education corresponds with a considerable decrease in child poor health outcomes^(68,69)^. Poor water, sanitation and hygiene facilities may carry serious early-life health consequences, as environmental contamination may increase disease prevalence, reduce nutrient absorption and lead therefore to chronic undernutrition^(70)^.

For Roma children, as for many other disadvantaged children living in high-risk environments, parental investment might be an important factor buffering against weight faltering, wasting and poor developmental outcomes^(71–73)^. Early development of Roma children was positively influenced by maternal investment; across various studies, activities with children such as reading, singing and playing are consistently associated positively with language, cognitive and socio-emotional development^(35)^. As in other studies, overall parental investment was low but higher for mothers^(30,74)^ and lowest for children born to mothers who married at 10–15 years of age, indicating that the overall investment received may be reduced in households with numerous children.

## Conclusion

The current study found no statistical evidence for an association between maternal age at first marriage and compromised child nutrition and development. Rather, the findings point towards other factors as the underlying essential determinants of child well-being among the Roma^(75)^. Among Roma, in addition to socio-economic status, child characteristics, maternal reproductive behaviour and parental investment were also shown to affect their children’s nutritional and developmental outcomes. Roma reproductive behaviour and parenting practices are shaped predominantly by cultural factors, whereas early marriage and early childbearing are often co-incident^(76)^. In Roma families, close and distant kin often serve as ‘helpers at the nest’, thus having supportive kin may not only be beneficial for reproductive success but also for children’s health^(65,77)^.

The current study possessed a number of limitations: the study design was cross-sectional, thereby limiting causal inference. The MICS data sets provide a limited range of mostly self-reported variables, thus allowing for potential biases. Other potential confounders, such as mother’s height, health status, history of preterm births or low birth weights of older children and levels of kin support, were not collected. The data collected were only for surviving children; inclusion of child mortality data would have affected the results obtained^(30)^. In light of these results, future research should focus upon investigating the many other factors that may possibly reveal associations between maternal age at marriage and children’s health and developmental outcomes.

Despite these limitations, the current study is the first to examine the association between maternal early marriage, child nutritional outcomes and early development using the Serbian Roma national data set, thus contributing to the literature on child outcomes in poor ethnic minority populations.
